# Swimming Activity Prevents the Unloading Induced Loss of Bone Mass, Architecture, and Strength in Rats

**DOI:** 10.1155/2015/507848

**Published:** 2015-05-18

**Authors:** Maurício J. Falcai, Ariane Zamarioli, Graziela Bianchi Leoni, Manoel Damião de Sousa Neto, Jose B. Volpon

**Affiliations:** ^1^Department of Biomechanics, Medicine and Rehabilitation, School of Medicine of Ribeirão Preto, University of São Paulo, 14049-900 Ribeirão Preto, SP, Brazil; ^2^Department of Restorative Dentistry, Dental School of Ribeirão Preto, University of São Paulo, 14049-900 Ribeirão Preto, SP, Brazil

## Abstract

We investigated whether swimming activity associated with a three-week period of hypoactivity could prevent the deleterious effects of disuse on the tibias of tail-suspended rats. Forty Wistar rats were divided into five groups: (HS) permanently hindlimb suspension rats; (HS + Swim) rats submitted to unloading interrupted by swimming exercise; (HS + WB) hindlimb suspension rats with interruption for regular weight bearing for the same length of time as the HS+Swim rats; (Control) control rats that were allowed regular cage activities; and (Control + Swim) control rats that underwent swimming exercise. At the end of the experiment, bone mineral density, bone strength, and trabecular quantification were analyzed. The hindlimb-suspended rats exhibited bone quality loss (significant decrease in BMD, bone strength, and deterioration of trabecular and cortical bone architecture; decrease in BV/TV, TbN, TbTh, ConnD, CtV, and CtTh; and increase in TbSp) when compared to control rats. In contrast, trained rats showed a significant increase of 43% in bone mass, 29% in bone strength, 58% in trabecular thickness, 85% in bone volume, 27% in trabeculae number, and 30% in cortical volume, when compared to the hindlimb-suspended rats. We conclude that swimming activity not only ameliorates but also fully prevents the deleterious effects on bone quality in osteopenic rats.

## 1. Introduction

Rat hindlimb suspension was developed as a model to study the effects of hypogravity, experienced by astronauts during space flight, on several organs and systems [1]. Later, the model was adapted to investigate bone deterioration secondary to disuse [2–4]. In humans, regardless of the cause, bone decay starts as an osteopenic state that can evolve into a final state of osteoporosis, which is characterized by increased bone mineral loss, very low density, and diminished bone strength, leading to a high fracture risk [5]. Bone fractures reduce life quality and increase mortality rates, especially in the elderly population [3, 5].

Several studies have shown that hypoactivity and the absence of weight bearing are closely associated with poor bone quality [2, 4, 6–9]. As a result, exercise, antiresorptive drugs, and other techniques, such as electrical muscle stimulation, have been proposed as therapies to improve or prevent bone deterioration [10–17]. Rubin et al. studied the effects of periods of unloading, interrupted either by regular weight bearing or vibration therapy, on the bone quality of rats. The authors found that vibration therapy, but not weight bearing, attenuated bone loss secondary to disuse [14]. Ju et al. observed that jump exercise intercalated with skeletal unloading showed a positive effect on the bone suppression of tail suspension-induced osteopenia in rats, by completely preserving bone deterioration due to unloading [10]. However, which type or regimen of muscular exercise would contribute more to restoring or preventing poor bone quality remains unclear, based on the divergent reports in the literature [18–20].

In this study, we investigated whether swimming activities associated with a three-week period of interrupted hypoactivity could prevent the deleterious effects of disuse on the tibias of tail-suspended rats. In addition, we assessed the effects of swimming on the bone quality of weight-bearing control rats.

## 2. Methods

### 2.1. Animals and Postoperative Care

Forty male Wistar rats (*Rattus norvegicus albinus*; body mass: 210 ± 10 g) were used in this study and were kept under standard laboratory conditions (room temperature 22 ± 2°C, humidity 55 ± 5%, 12 h light-dark cycles). Young adult rats were fed unrestrictedly with standard laboratory animal chow, containing 1.15% calcium and 0.88% phosphorus; they were offered water* ad libitum*. The experimental protocol and all of the animal care described herein were previously approved by the Animal Care and Use Committee of our Institution. After one week of acclimatization, the animals were randomly assigned to one of five groups (*n* = 8 per group): (1) hindlimb suspension rats (HS): permanently unloaded rats; (2) HS + swimming (HS + Swim): rats submitted to unloading interrupted by swimming exercise periods; (3) HS + weight bearing (HS + WB): rats submitted to hindlimb suspension, with interruptions for regular weight bearing for the same length of time as the HS + Swim rats; (4) control (Control): control rats that were not submitted to unloading and were allowed to continue their regular cage activities; and (5) control-swimming (Control + Swim): control rats that were not submitted to unloading and that underwent swimming exercise periods.

### 2.2. Hindlimb Suspension

**Figure 1:**
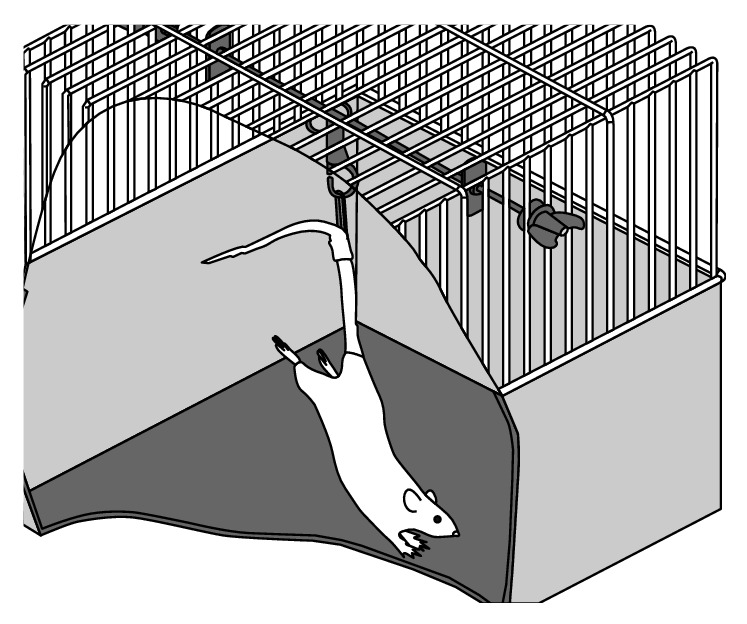
Illustrative image of the tail suspension method.

The rats were suspended by their tails using a procedure and equipment very similar to those described by Morey-Holton and Globus (2002) [7]. In brief, the tails were sprayed with a 20% tincture of benzoin (Styrax spp.; Rioquimica, Rio de Janeiro, RJ, Brazil). Then, a single strip of Skin-Trac tape (Reston 1560 M, 3 M, Sumare, SP, Brazil) was affixed to the sides of the tail so that it formed a loop at the tip of the tail. The loop was attached to a swivel, and an overhead trolley system was created using a transversal metallic bar attached to a higher level (Figure 1). The height of the suspending system and the range of trolley movement were adjusted, so the animal trunk was kept at a 30° of inclination, thus allowing the rat to move around in the cage with the forelimbs bearing weight and with it having to reach for water and food. The hindlimbs, in contrast, were not allowed to touch either the cage bottom or the cage walls. The rats were suspended for 21 consecutive days and were monitored for signs of stress, tail circulatory embarrassment, or skin wounds caused by the suspension system. The tail suspension was only interrupted to allow the swimming activities and the regular weight bearing in the HS + Swim and HS + WB groups, respectively, for one hour five times per week, as defined in the specific protocols. Iodine was applied to the animals' tails to discourage their chewing of the harness.

### 2.3. Swimming

Animals from the HS + Swim and Control + Swim groups were submitted to the exercise training of swimming over three weeks, five days per week for 60 minutes per day. Training was performed in a tank 100 cm long, 60 cm wide, and 60 cm deep, with a water level of 40 cm and a temperature of 31 ± 1°C. Training began on day one after hindlimb suspension. The training protocol was conducted during the same period of the day in all of the training sessions. The first week of swimming consisted of adaptation training, with the rats swimming for 15 minutes at gradual increments until they reached 60 minutes by the end of the first week (a total of 15 sessions). Weights were not attached to the animals.

For animals from the HS + WB group, unloading was interrupted, and cage weight bearing was allowed for the same amount of time as in the swimming training protocols of animals from the HS + Swim and Control + Swim groups. Throughout the free cage activities (60 minutes), the HS + WB rats were carefully monitored so that they were not siting during this period instead of bearing weight.

After 21 days of suspension/experimentation, the animals were euthanized with an excessive dosage of anesthesia (pentobarbital). The right tibias were then dissected, cleaned of soft tissues, and analyzed by micro-CT (a nondestructive analysis), followed by destructive whole-bone mechanical testing.

### 2.4. Micro-CT Analysis: BMD and Microstructure Assessment

A high-resolution, desktop micro-CT system (SkyScan 1174v2; Bruker-microCT, Kontich, Belgium) was used to quantify the BMD and the three-dimensional microarchitecture parameters in the proximal tibia. The specimens were scanned using 50 kV and 800 mA, with the aid of a 0.5 mm thick aluminum filter to optimize the contrast, a rotation step of 1.2°, two-frame averaging, and an isotropic resolution of 13.9 *μ*m. Images of each specimen were reconstructed with dedicated software (NRecon version 1.6.3; Bruker-microCT), providing axial cross sections of the inner structures of the samples. Two regions of interest were made, one at the tibial proximal metaphysis, which mainly contains trabecular bone, and another at the mid-diaphysis, which contains mainly cortical bone. The reconstruction of the proximal metaphysis was selected manually starting just distally of the growth plate for an extension of 3 mm. The reconstruction of the diaphysis was defined by a 2 mm region starting 6 mm distally from the growth plate. CTAn software (Bruker-microCT), version 2.2.1, was used for the determination of the optimal threshold from the image histograms and was set to exclude soft tissue but to include poorly mineralized bone. The same threshold was used in all of the samples. The thresholded image was used as a mask to measure the BMD of the bone structures. For the accurate calculation of BMD, appropriate calibration of the Skyscan CT analyzer was performed with known density calcium hydroxyapatite phantoms (0.25 and 0.75 g/cm^3^). Once the phantoms' BMDs were calibrated in the CTAn software, a volume of interest (VOI) of 3 mm was selected in the bone. Trabecular architecture of the proximal metaphysis was characterized by determining trabecular bone volume fraction (BV/TV), trabecular number (TbN), trabecular thickness (TbTh), trabecular separation (TbSp), and density of connectivity (ConnD). Cortical architecture was assessed in the diaphysis and was characterized by cortical volume (CtV) and cortical thickness (CtTh). All bone morphometric measurements and nomenclature are in accordance with recommendations of the ASBMR [21].

### 2.5. Mechanical Testing

The mechanical properties of the bones were determined by testing them to fracture using a mechanical testing device (EMIC, PR, Brazil) equipped with a 500 N load cell. The isolated tibia (fibula was removed), with the anterior surface facing up, was placed on two metallic supports 25 mm apart. The load was vertically applied at a constant displacement rate of 1 mm/min at the mid-diaphysis. The load-deflection curve was obtained in real time, and maximal load and load at the yield point were assessed (TESC software, version 13.4) to represent the general bone integrity and the strength within the elastic phase [22]. The bones were tested by the same technician, who was unaware of the identity of the bones.

### 2.6. Data Analysis

All of the data are expressed as the means ± standard deviations. All of the statistical analyses were carried out with the IBM SPSS Statistics version 20 (Armonk, NY, USA). Comparisons among the groups were statistically processed by nonparametric Kruskal-Wallis (ANOVA), followed by Dunn's* post hoc* analysis. The level of statistical significance was set at *P* < 0.05.

## 3. Results

### 3.1. BMD of the Tibia

**Figure 2:**
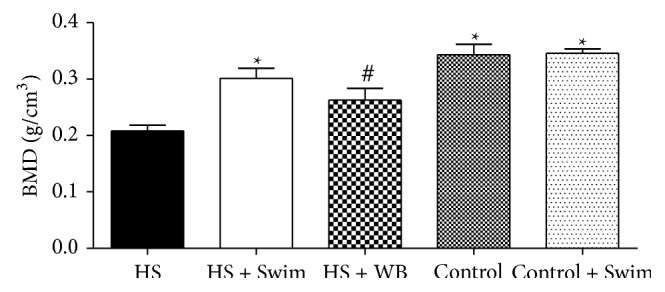
Comparison of the BMD in the proximal tibia shows a significant decrease in bone mass in the unloaded rats (HS versus Control, *P* < 0.001), which was completely preserved by swimming exercises (HS versus HS + Swim, *P* < 0.01; HS + Swim versus Control, *P* > 0.05). Different signs indicate significant difference (*P* < 0.05).

After three weeks of tail suspension, the areal proximal tibial BMD in the hindlimb-suspended group had 38% lower values, compared with the control rats (0.34 g/cm^3^ versus 0.21 g/cm^3^, *P* < 0.001) (Figure 2). In contrast, swimming exercise during the hindlimb unloading period (HS + Swim) resulted in significantly higher values of tibial BMD, compared with the HS group (+43%, *P* < 0.001). Swimming exercise not only ameliorated bone mass in the HS + Swim rats but also completely reverted its value to those considered normal (HS + Swim versus Control and Control + Swim, *P* > 0.05). Although weight bearing lonely increased BMD in the HS + WB group (+24% versus HS, *P* > 0.05), the difference was not significant. No significant difference was also found between the regular/sedentary control rats and control rats that swam (Control versus Control + Swim, *P* > 0.05).

### 3.2. Bone Strength of the Tibia

**Figure 3:**
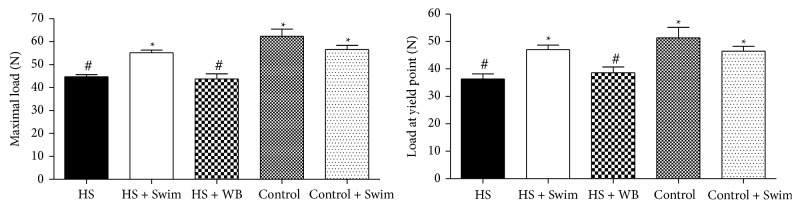
Comparison of the bone strength of the tibia shows a significant decrease in the maximal load in the unloaded rats (HS versus Control; *P* < 0.001), which was completely preserved by swimming exercises (HS versus HS + Swim, *P* < 0.01; HS + Swim versus Control, *P* > 0.05). Different signs indicate significant difference (*P* < 0.05).

After three weeks of tail suspension, the bone strength at the tibia in the unloaded group (HS) was 28.5% weaker (measured by maximal load and load at the yield point) than that of the bones from the control rats (44.7 N versus 62.3 N for maximal load and 36.4 N versus 51.3 N for load at the yield point, *P* < 0.001) (Figure 3). In contrast, swimming exercise during the hindlimb unloading period (HS + Swim) resulted in significantly higher values of tibial maximal load, compared not only with the HS group (+23%, *P* < 0.001, 55.1 N versus 44.7 N) but also with the HS + WB group (+26%, *P* < 0.01, 55.1 N versus 43.7 N), whence swimming exercise completely preserved bone strength (HS + Swim versus Control, *P* > 0.05). With regard to the load at the yield point, swimming (HS + Swim), but not regular weight bearing (HS + WB), increased bone strength in +29%, when compared to the HS rats (47.02 N versus 36.36 N, *P* < 0.05). No significant differences were found between the control rats that swam and the sedentary controls (Control versus Control + Swim, *P* > 0.05).

### 3.3. Microstructural Properties

#### 3.3.1. Trabecular Architecture

**Figure 4:**
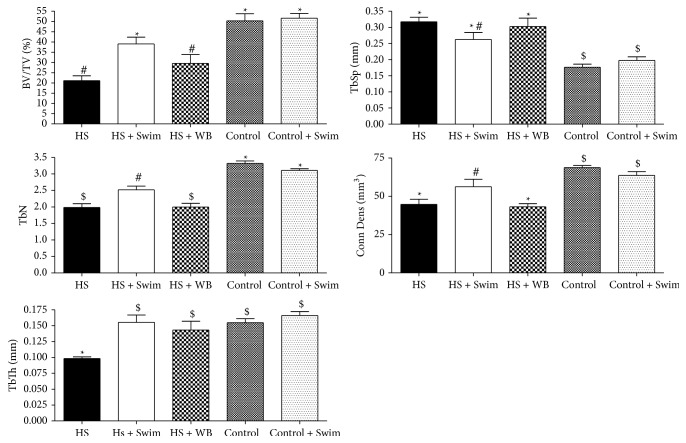
Unloading caused a significant decrease in the bone volume ratio (BV/TV), trabeculae number (TbN), trabeculae thickness (TbTh), and density of connectivity (Conn Dens) at the proximal tibias of hindlimb-suspended rats when compared to the controls (HS versus Control, *P* < 0.05). BV/TV was partially preserved by swimming exercises (HS + Swim versus HS, *P* < 0.05; HS + Swim versus HS + WB, *P* < 0.05) but not by regular weight bearing (HS versus HS + WB, *P* > 0.05). TbN was partially preserved by swimming exercises (HS + Swim versus HS, *P* < 0.05; HS + Swim versus HS + WB, *P* < 0.05) but not by regular weight bearing (HS versus HS + WB, *P* > 0.05). Conn Dens was completely preserved in both the swimming and regular weight-bearing groups (*P* < 0.01 versus HS; *P* > 0.05 versus Control/Control + Swim). Different signs indicate significant difference (*P* < 0.05).

Three weeks of unloading by hindlimb suspension induced a marked deterioration of the trabecular architecture in the proximal metaphysis of the tibia (58% decrease in the BV/TV, 40% decrease in trabeculae number, 37% decrease in thickness, and 38% decrease in density connectivity, *P* < 0.01, Figure 4), compared with control rats. Both swimming (HS + Swim) and regular weight-bearing activities (HS + WB) were efficient at increasing trabecular thickness (+58% and +46%, resp., *P* < 0.001, Figure 4), whereas the other microtrabecular parameters were improved due to the swimming (HS + Swim) activities alone (85% in BV/TV and, 27% in TbN, *P* < 0.05, Figure 4), when compared to the HS rats. Although swimming showed positive effects on the unloaded bones, it did not improve the microarchitecture in healthy bones under regular loading (Control versus Control + Swim, Figure 4).

#### 3.3.2. Cortical Architecture

**Figure 5:**
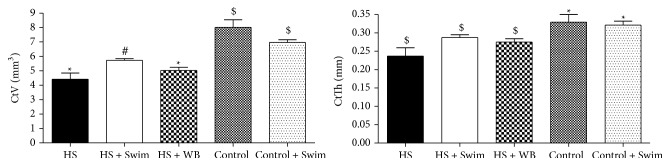
Unloading caused a significant decrease in the cortical volume (CtV) and cortical thickness (CtTh), at the diaphysis of tibias of hindlimb-suspended rats when compared to the controls (HS versus Control; *P* < 0.05). CtV was partially preserved by swimming exercises (HS + Swim versus HS, *P* < 0.05; HS + Swim versus HS + WB, *P* < 0.05) but not by regular weight bearing (HS versus HS + WB, *P* > 0.05). Different signs indicate significant difference (*P* < 0.05).

Three weeks of unloading by hindlimb suspension also induced deleterious changes in the cortical bone of the diaphysis of the tibias, but to a lesser degree than the losses in the trabecular architecture of the proximal metaphysis (−45% decrease in the CtV and −28% in CtTh, *P* < 0.01, Figure 5), compared with control rats. The suspended rats submitted to swimming exercise (HS + Swim) showed higher cortical volume than the hindlimb suspended (HS) only (+30%, *P* < 0.05), whereas cortical bone from the nonsuspended but swimmer rats did not differ from the control rats (Control + Swim versus Control, Figure 5).

**Figure 6:**
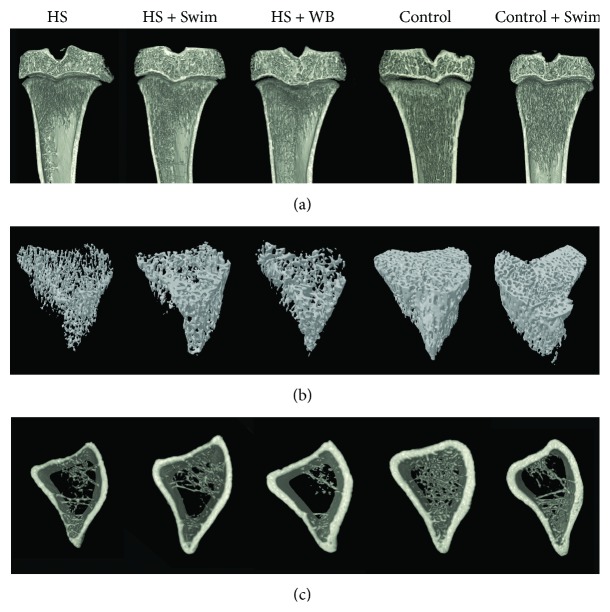
Microarchitectural images of the proximal tibias show fewer and thinner trabeculae in the hindlimb-suspended rats (HS), which were partially preserved by swimming activities (HS + Swim).

Figure 6 shows three-dimensional images of the bone microarchitecture in tibias.  Figure 6(a) consists in a coronal section of proximal tibia, Figure 6(b) presents only trabecular bone in the metaphysis of proximal tibia, and Figure 6(c) shows cross-sectional images of cortical bone in the diaphysis of tibias. Unloading due to hindlimb suspension reduced the amount, thickness, and connectivity of the trabeculae, compared to the control animals. Similarly, unloading (HS) decreased volume and thickness of cortical bone. Swimming (HS + Swim) and weight bearing (HS + WB) partially preserved the bone loss microarchitecture, whereas the changes were more pronounced in the HS + Swim rats.

## 4. Discussion

Osteoporosis due to disuse has been assessed in human studies mainly by two models, bed rest and microgravity as experienced during space flights [23, 24]. Experimental studies were performed in rats that were submitted to space flights to study the effects of microgravity exposure. However, data interpretation and comparison with matched groups have been challenging due to the limited sample sizes and large variety with regard to the time of exposure to microgravity and dietary disparities [24]. Thus, hindlimb suspension has been used in the scientific literature as a valid experimental model to simulate the effects of hypogravity [25]. This approach has the advantage of low financial cost, and it is well suited to methodological standardization. In our study, we used hindlimb suspension in rats as a model to assess disuse osteopenia caused by hypoactivity and the absence of weight bearing [1–4]. The loss of bone volume and quality due to the lack of mechanical stimuli has been recognized and explained since 1870 by Wolff's Law [26]. This theory states that a bone will adapt to the load under which it is placed [27]. Thus, a bone that is not submitted to mechanical stimuli undergoes an increased bone resorption activity resulting in site-specific bone loss, as the events related to mechanotransduction are not fully active. Mechanotransduction is the conversion of physical signals into biochemical response, which results in altered gene expression, cell function and morphology, and extracellular matrix formation [28]. Our results show that hindlimb suspension deteriorates bone quality at significantly decreasing bone mass, strength, and microarchitecture, which suggests the involvement of bone cells related to the mechanotransduction. Osteocytes are bone cells that detect the mechanical signals and modulate bone formation and resorption by signaling osteoblasts and osteoclasts [28]. Thus, we believe that the absence of weight bearing and hypoactivity in our hindlimb suspended rats caused an unbalanced bone turnover, leading to bone loss.

Weight-bearing exercises and muscle contractions have been used to study the effects of these techniques on the bone tissue quality of osteopenic rats [13, 15, 16]. It is well known that mechanical stimuli play important positive roles in osteogenesis through a still poorly understood mechanism called mechanotransduction. Donahue et al. demonstrated that annexin V (AnxV) was a mechanoreceptor that increased intracellular calcium and thus was associated with bone formation [29]. Although mechanical loading by means of weight-bearing exercises has been widely recognized to improve bone mass, the effects of isolated muscle contractions through non-weight-bearing exercises on bone metabolism remain inconclusive. Tenforde and Fredericson showed that while weight-bearing sports improved bone mass, swimming could negatively affect bone tissue [19]. In agreement with this report, Scofield and Hecht showed that adolescents and adults who participated in non-weight-bearing sports (e.g., swimming) often had lower bone mineral density than athletes participating in weight-bearing sports (e.g., running) [18]. In contrast, previous studies have shown an osteogenic effect of muscle contraction in the absence of weight-bearing conditions in rats, such as electrical stimulation while their tails are suspended, or when the rats were under paraplegic conditions [10, 13, 14, 30, 31]. In our study muscle contraction during swimming activity significantly improved bone quality, suggesting that bone tissue responds to mechanical loading by stimulating bone formation where strain is higher [22]. Due to the buoyancy of water, the osteogenic effect observed in swim-exercised rats is derived primarily from the mechanical loading generated by muscle contraction. Additionally, in spite of increasing bone formation, mechanical stimuli also reduce bone resorption, highlighting the unique and potential osteogenic effect of the physical exercise as an antiosteopenic therapy. Differently than antiresorptive drugs, mechanical stimulus releases both prostaglandins and nitric oxide from bone cells and so induces an increase in bone formation and a decrease in bone resorption. Conversely, antiresorptive drugs increase both bone formation and resorption [22]. Thus, we believe that several pathways involved in the mechanotransduction were signaled by the mechanical stimuli and either stimulated bone formation or downregulated bone resorption. Among these pathways we may cite the inositol 1,4,5-triphosphate pathway, which causes an increased intracellular calcium in osteoblastic cells soon after the mechanical stimulus begins and is necessary for the expression of bone matrix proteins and the Wingless-type signaling through the Lrcp5 receptor, which responds to mechanical stimuli and is related to the expression of osteopontin, a bone matrix protein. Insulin-like growth factors (IGF) have also been recognized as a necessary mediator of mechanically induced bone formation. Hormones, such as the estrogen and the parathyroid hormone, also play an important role in enhancing the osteogenic effect of mechanical stimuli, by increasing bone formation [22].

Few limitations may be highlighted within this study. Our data cannot be directly extrapolated to clinical practice due to the difficulty to accurately establish whether the rat's swimming mechanism is exactly the same as that in humans. In humans, swimming has been widely studied as a sport modality, but its effects as a preventive tool for bone loss have not been entirely elucidated in the literature. In this regard, studies with humans are necessary to establish the best swimming program to be applied to each patient when taking into account individual profiles. Therefore, molecular assessments of genes and proteins related to disuse bone loss and mechanotransduction cells were not performed in this study. However, we speculate that several pathways may be involved in the increased bone mass and strength and in the improved trabecular and cortical microarchitecture (i.e., AnxV, inositol 1,4,5-triphosphate pathway, Wingless-type signaling, and IGF). In fact, we focused on the assessment of bone quality encompassed by body mass, mechanical resistance, and bone microarchitecture.

In our study, we found that regular weight bearing slightly ameliorated bone loss in unloaded animals, whereas swimming exercises acted as a potent osteogenic tool in stimulating bone formation. Swimming activities not only improved bone loss in unloaded rats but they also completely preserved the bone mass and bone strength of these animals, restoring the bone mineral density and maximal load values to levels similar to those found in the control rats. This finding shows that the mechanical stimuli provided by the muscle contractions due to swimming activity played an important role in the protection of bone tissue against decreases in bone quality. In contrast, swimming failed to improve bone mass in regular weight-bearing rats. Thus, we believe that, to increase bone mass in healthy bones, mechanical loading must be present by means of weight bearing.

## 5. Conclusions

We conclude that swimming activity not only ameliorates but also fully prevents the deleterious effects on bone quality in osteopenic rats. Thus, muscle contraction provided by the swimming activity was effective at preventing the deleterious effects of unloading on bone mass, strength, trabecular, and cortical architecture, confirming the osteogenic role of mechanical stimuli on bone formation. Further studies should assess the effects of swimming on the bone quality in individuals with bone loss due to disuse.
